# Archetypal Analysis Reveals Consistent Visual Field Patterns for Stimulus Size III and Size Modulation Perimetry in Glaucoma

**DOI:** 10.1167/tvst.14.10.12

**Published:** 2025-10-10

**Authors:** David Szanto, Edward Linton, Michael Wall

**Affiliations:** 1 Renaissance School of Medicine, Stony Brook, NY, USA; 2 Department of Ophthalmology, New York Eye and Ear Infirmary, New York, NY, USA; 3 Iowa City VA Center for the Prevention and Treatment of Visual Loss, Iowa City, IA, USA; 4 Department of Ophthalmology and Visual Sciences, University of Iowa, Iowa City, IA, USA

**Keywords:** Visual Field, Size Modulation Perimetry, Standard Automated Perimetry

## Abstract

**Purpose:**

Optic nerve disorders produce identifiable visual field (VF) patterns. Size modulation perimetry (SMP) unlike standard automated perimetry (SAP) shows little degradation of retest variability with increasing VF damage. We hypothesize that VF testing with stimulus size III (size III) and SMP would reveal analogous patterns, supporting interchangeability in clinical practice.

**Methods:**

We analyzed 274 same-day pairs of VFs from 83 eyes of patients with glaucoma. Participants underwent testing every 6 months using size III and SMP VFs. We converted raw sensitivity data to total deviation (TD) scores relative to normative datasets, censoring size III sensitivities below 20 decibels (dB). Archetypal analysis (AA) was applied separately to each modality to identify characteristic VF patterns, or archetypes (ATs). We evaluated both absolute VF patterns and longitudinal VF changes by subtracting baseline measurements. Cosine similarity measured the overlap between ATs in size III and SMP.

**Results:**

Absolute size III and SMP VFs exhibited overlapping patterns, with 95% of ATs demonstrating a cosine similarity of ≥0.50. Change ATs showed lower similarity scores (30% with cosine similarity ≥0.50). However, some ATs still revealed similar underlying defects.

**Conclusions:**

AA consistently extracted comparable VF defect patterns from size III and SMP in glaucoma. Absolute size III patterns closely aligned with SMP, whereas patterns of change showed partial agreement.

**Translational Relevance:**

These findings suggest that SAP and SMP capture similar patterns of VF loss.

## Introduction

Standard automated perimetry (SAP) using a stimulus size III (size III) with a 24-2 visual field (VF) test is a widely used method for evaluating visual function, particularly in conditions like glaucoma. This type of VF measures visual sensitivity with a point of constant radius (4 mm^2^, 0.43 degrees) with varying luminance.^1^ The 24-2 VF test assesses sensitivity at 54 test locations within the central 24 degrees of vision, spaced 6 degrees apart, providing a map of functional vision loss. Glaucoma causes progressive loss of retinal ganglion cells, resulting in a variety of VF deficit patterns that correspond to damage in retinal nerve fiber layer (RNFL) bundles.^2^^,^^3^ SAP is an essential tool for diagnosing and monitoring optic neuropathies like glaucoma. However, when using the conventional size III stimulus, SAP has a significant limitation where test-retest variability increases as VF damage progresses.^4^

One leading theory for why SAP struggles with variability in locations with significant damage is that, as ganglion cells are lost with VF damage, cortical receptive fields develop gaps where a stimulus is less likely to generate a response. When testing with a small, fixed-size stimulus such as size III, these “holes” may lead to inconsistent responses, as the stimulus may cover an area with few or no receptive fields and not be seen on one trial but cover a critical number of receptive fields on another trial and be seen.^5^

This “Swiss-cheese” sampling-loss model is supported by repeatability studies showing that test-retest variability for size III rises steeply below about 20 decibels (dB)^4^^,^^6^ by modeling work demonstrating that such excess variability can be explained by structural undersampling of surviving receptive fields,^7^ and by spatial summation studies linking local ganglion-cell loss to gaps within enlarged cortical receptive fields in glaucoma.^8^ Further, microfixation shifts result in the stimulus falling on different numbers of receptive fields. The spatial averaging effect of the larger stimulus helps to fill in more of these gaps, and due to the microfixation shifts, there is reduced variability in the number of receptive fields being stimulated.^9^

This results in increased test-retest variability, making it difficult to determine whether changes in sensitivity are due to true disease progression or perimetric noise. For SAP with size III stimuli, retinal locations with sensitivities below about 20 dB are difficult to measure, as the variability on retest is extreme.

Larger stimulus sizes, such as stimulus size V (which has a stimulus 16 times the area of size III) and VI (64 times the area of size III), help overcome the first hypothesis by covering many more receptive fields in damaged areas. This promotes improved space averaging and makes detection more stable and improves test-retest repeatability.^10^ However, this comes at the cost of reduced spatial resolution.

Censoring a field by setting all sensitivity values below a specific threshold to that threshold value can minimize measurement noise.^4^ Setting a predefined cutoff ensures that unreliable data points do not distort statistical analyses or clinical interpretations. This method has been particularly useful when comparing VF tests obtained with different stimulus sizes.^11^

Size modulation perimetry (SMP) is similar to SAP, but measures sensitivity by varying the area of the stimulus instead of the luminance. The strategy can be thought of as trying to find the smallest area that contains a critical number of ganglion cell receptive fields, rather than trying to quantify the density of receptive fields present in a given area. This technique minimizes test-retest variability by determining the smallest stimulus size required for detection, making it particularly valuable in conditions where conventional perimetry may yield inconsistent results.^12^^–^^14^

SMP (also termed area modulation or size threshold perimetry elsewhere in the literature) has been shown to have stable test-retest variability, without an increase in damaged areas. The majority of evaluations of SMP in the literature have dealt with individual test locations and little has been written about patterns of VF loss.

SMP is particularly well-suited for detecting changes in Ricco’s area, or the maximal region over which complete spatial summation occurs, where sensitivity increases with stimulus size up to a critical point. In glaucoma, Ricco's area is known to expand as retinal ganglion cells are lost.^8^^,^^15^ Because SMP increases the stimulus area until detection occurs, it adjusts to this expansion and allows thresholds to be measured under complete summation. This adaptive behavior helps maintain low test-retest variability across the VF and potentially improves the signal-to-noise ratio in damaged regions.^15^^,^^16^

Swanson et al. and Redmond et al. report that Ricco's area itself enlarges as ganglion cells are lost, implying that cortical summation pools a constant ganglion cell count at the cost of spatial resolution.^7^^,^^8^ Taken together, current data suggest a continuum: receptive-field enlargement maintains sensitivity early in disease, but as ganglion cell density declines further true sampling gaps may emerge once it falls below a critical level. These mechanisms are not mutually exclusive – an enlarged cortical field can still contain “holes,” particularly when using small stimuli that risks undersampling. Prospective studies that pair adaptive-area stimuli with high-resolution retinal imaging should clarify where these mechanisms cross over and guide the stimulus design for each disease stage.

The Variability in Perimetry II study was a prospective veterans affairs (VA)-funded study that enrolled healthy observers and glaucoma subjects and measured VF sensitivity using a custom SMP test, alongside SAP with sizes III, V, and VI. The dataset generated from this study provides a unique opportunity to compare SMP and SAP to determine whether patterns of the RNFL bundle loss are similarly revealed by each strategy.^13^

Archetypal analysis (AA) is a dimensionality reduction technique that has been applied for unsupervised VF analysis. The algorithm identifies extremal points within a dataset, known as archetypes (ATs), allowing each data point to be represented as a weighted combination of these ATs.^17^ AA is particularly effective in identifying and quantifying recurrent patterns within datasets and has been widely applied to detect characteristic VF loss patterns in both 24-2 and 10-2 testing.^18^^–^^25^

To our knowledge, no study exists comparing the AT patterns of SAP with SMP. We hypothesize that VFs obtained with size III and SMP VFs will show similar AT patterns as well as similar changes in VF patterns over time in glaucoma. If successful, clinicians could confidently utilize either SAP or SMP based on the specific clinical context. This flexibility would be particularly valuable in cases of severe vision loss, whereas SMP excels by not having substantial increases in variability with increasing damage and thereby providing more reliable measurements.

## Methods

This study was approved by the Institutional Review Board of the University of Iowa and required no additional consent as the data used were de-identified and from participants who had consented for use of their data. The study was conducted according to the tenets of the Declaration of Helsinki.

VF data using size III and SMP were obtained from the Variability in Perimetry II (VIPII) study, which examined differences in variability across perimetric stimulus sizes and their effectiveness in distinguishing between healthy and glaucomatous VFs. It was a prospective study which was conducted with rigorous standardization of the VF testing protocol and strict quality control. Variability in this study was low, relative to other published studies.^6^^,^^13^ This study analyzed participants with moderate to severe VF loss due to glaucoma, who underwent same-day VF testing with both standard 24-2 SITA Standard using Goldmann size III stimuli and SMP, at the University of Iowa Department of Ophthalmology and Visual Sciences. SMP used a staircase procedure to determine a size threshold, defined as the smallest size stimulus seen by the subject at each test location where the subject made a response meeting prespecified criteria. The stimulus was a light gray filled circular patch of 80 apostilbs; the background was a uniform (nonstippled) darker gray with a luminance of 50 apostilbs. Stimuli were presented in 18 sizes, with a diameter step factor of 10^1^. The diameter ranged from 0.13 degrees to 8.46 degrees. A trial consisted of three steps: the stimulus was displayed, the subject touched a light pen to the location where the target was perceived, and feedback was given with a crosshair showing the location of the touch relative to the target (a nonfilled circle). A 2/1 staircase procedure was used to estimate the threshold. The SMP test grid included 44 points aligned with the 24-2 layout, omitting the top and bottom rows (±21 degrees) and the 2 far-nasal locations (x = 27 degrees). The order of SAP and SMP testing was randomized at each visit as part of the VIPII protocol, and all participants received ≥5-minute rest breaks between tests to reduce fatigue. Inclusion criteria required glaucomatous optic disc changes with abnormal SAP and a diagnosis of primary, secondary, or normal-tension glaucoma, with no other vision-affecting conditions. Exclusion criteria included cataracts reducing visual acuity below 20/30, pupil size less than 2.5 mm, age under 19 years, or pregnancy. We required either good gaze tracking or fixation monitoring by the perimetrist because pseudo-fixation losses from head tilts are not uncommon. If the subject was not a good perimetry subject, they were excluded by the perimetrist.^13^

### Data Censoring and Conversion

We recorded raw sensitivity values for each test. Each participant had same-day VF testing of both size III and SMP VFs every 6 months for 4 years. We censored size III at 20 dB, where sensitivity values below this threshold were replaced with 20 dB to minimize measurement noise and improve comparability. Sensitivities from both size III and SMP were then age-corrected to 45 years, with a normative database for SAP and a derived reference set from VF data of 60 healthy participants tested twice with SMP. Sensitivities were then age-standardized and converted to total deviation (TD) values using the same datasets. To ensure a direct comparison between size III and SMP, we aligned test locations from SMP to its 24-2 counterparts while excluding the 10 additional peripheral points present in the 24-2 grid that SMP does not measure. All sensitivity values from same-day same-type VFs were averaged.

We conducted two separate analyses. The first utilized all available VFs from both size III and SMP to assess overall patterns and relationships between the two modalities, or absolute VF analysis (AVFA). The second focused on longitudinal changes, where each participant's first VF served as a baseline, and subsequent VFs were analyzed by applying pointwise subtraction from the baseline to quantify changes over time, or VF change analysis (VFCA). This approach generated change maps, with positive values indicating improvement and negative values indicating deterioration in TD units at specific test locations over time.

### Archetypal Analysis and Statistical Analysis

All statistical analyses were conducted using Python version 3.8.8, with AA performed using the “archetypes” module.^19^ To evaluate model performance, we computed the residual sum of squares (RSS) by comparing the reconstructed VF data to the original dataset across models containing 2 to 20 ATs.

To determine the optimal number of ATs, we generated plots for both size III and SMP to illustrate the relationship between RSS values and the number of ATs. The final number of ATs was selected using the elbow method, balancing the trade-off between minimizing RSS and limiting model complexity.

We normalized the sum of relative weights (RWs) across all ATs to 100%, ranking ATs by RWs to reflect their prevalence in the dataset. Each VF was then decomposed into a weighted combination of ATs, such that the AT weighting coefficients sum equaled 100% per VF.

To group similar ATs by VF type, we used cosine similarity, a metric used in VF analysis that quantifies the similarity between two VFs.^18^^,^^25^ This was achieved by computing the normalized dot product of their values, treating each VF as a 42-point vector (excluding the 2 blind spot points). Cosine similarity scores range from –1 to 1, with values closer to 1 indicating a high degree of similarity between patterns. A key advantage of cosine similarity is its independence from intensity. If TD values were doubled at every location, the similarity score would remain unchanged. This property is particularly important because ATs represent weighted combinations of VF patterns, allowing for comparisons based purely on shape rather than differences in defect severity.

We calculated the cosine similarity between every possible AT pair from size III and SMP. To ensure comprehensive comparisons, we generated two separate mapping approaches:

1. Listing the most similar SMP ATs for each size III AT (size III-SMP mapping).
2. Listing the most similar size III ATs for each SMP AT (SMP-size III mapping).

This dual approach was necessary because multiple ATs from one modality could be most similar to a single AT in the other modality. This way, we ensured that each pattern received adequate representation in terms of similarity, allowing for a more balanced and accurate comparison of VF loss patterns across stimulus types.

## Results

We analyzed 274 pairs of VFs from 83 eyes with glaucoma. The mean age was 66.7 ± 9.1 years. For the AVFA, the mean TD after censoring was –4.4 ± 2.1 dB and was –8.3 ± 4.7 dB without censoring for size III stimuli (29% points censored). SMP had a mean TD of –12.0 ± 5.8 dB. The VFCA used a total of 189 pairs of VFs with a mean TD change of –0.1 ± 0.8 dB for size III and 0.0 ± 1.7 dB for SMP.

**Figure 1.**
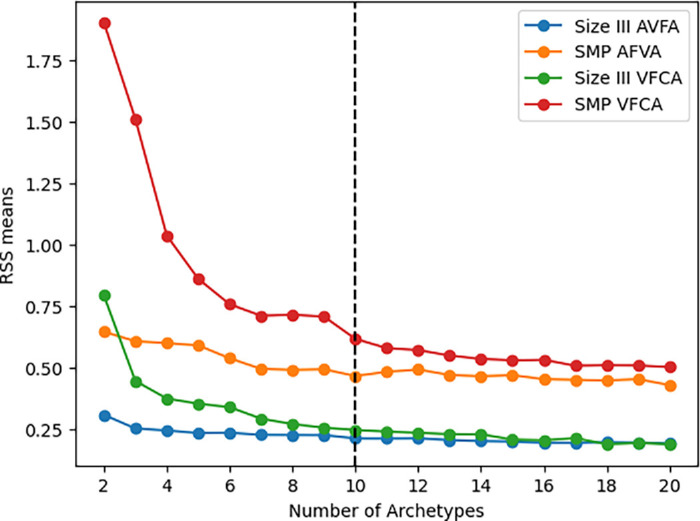
**RSS curves.** Plot of the residual sum of squares versus the number of archetypes to identify the optimal archetype count for visual fields using stimulus size III and size modulation perimetry in glaucoma. We selected 10 archetypes for all models, as the curve begins to plateau, striking a balance between predictive accuracy and overfitting.

Based on the RSS curves, we selected 10 ATs as this represented a local minimum near the global minimum while maintaining a relatively low AT count (Fig. 1). Although this may not be the strictly optimal number mathematically, it strikes a balance between capturing the major VF patterns and avoiding excessive complexity. As our goal is to map the major patterns from one VF type to another, we opted for a conservative approach to minimize the inclusion of noise. We also used 10 ATs for both analyses for consistency.

**Figure 2.**
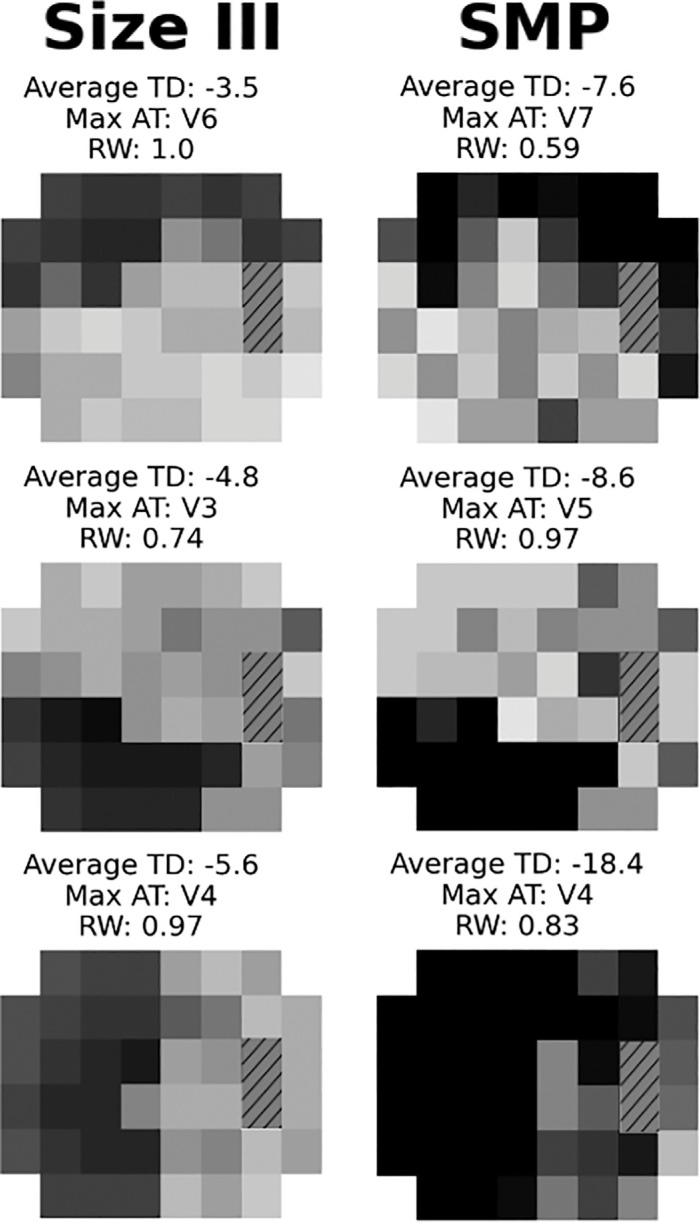
**Select decomposed visual fields.** Absolute visual fields from individual participants are shown, with darker shades indicating greater deficits. Each row represents a separate individual’s scan. The *left column* displays stimulus size III visual fields, whereas the *right column* presents the corresponding same-day size modulation perimetry scan, along with the average total deviation, dominant archetype, and its relative weight.

We generated AT maps for each VF and study type (Supplementary Figs. S1, S2, S3, S4). Comparisons of select absolute VFs by type and their corresponding AT breakdowns are shown (Fig. 2). We also generated absolute AT maps for size V and size VI (Supplementary Figs. S5, S6).

### Absolute Visual Field Analysis

**Figure 3.**
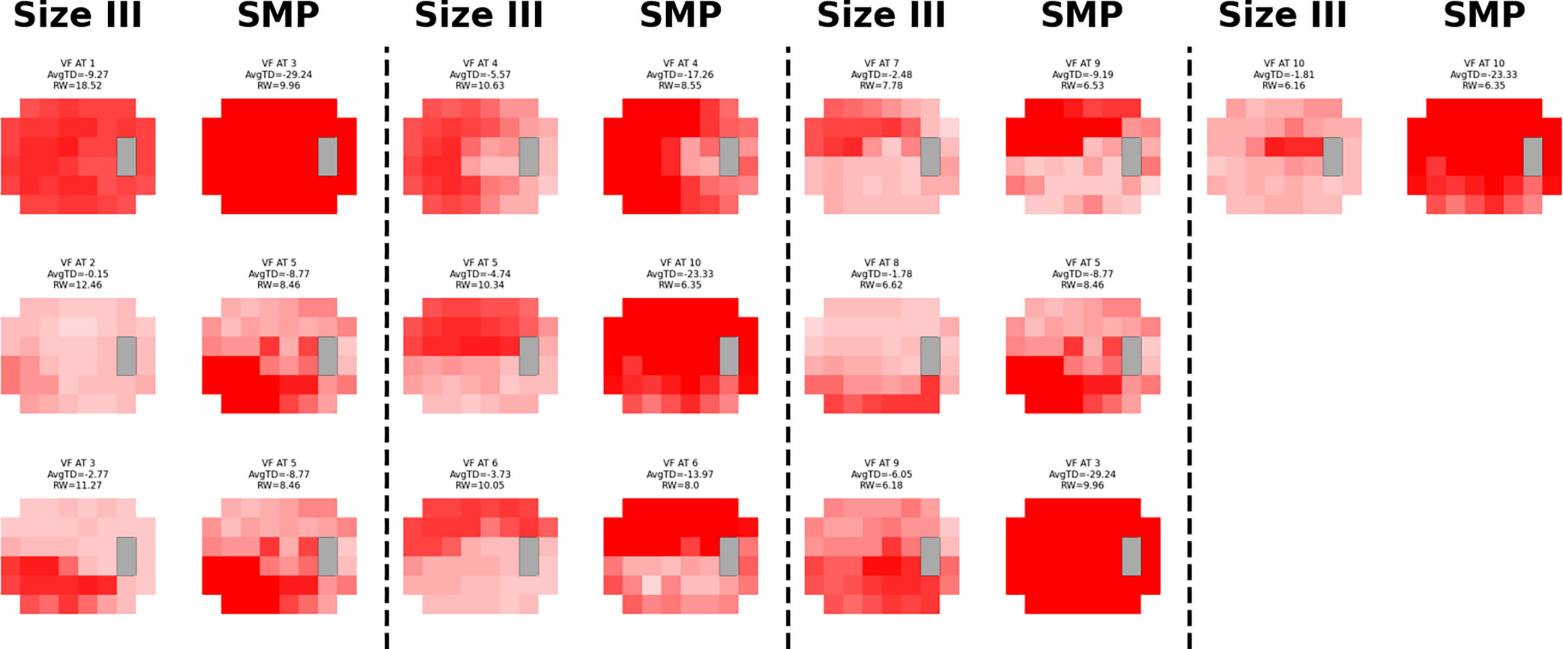
**AVFA size III-SMP mapping.** Visual field patterns for glaucoma using stimulus size III (*left*) and size modulation perimetry (*right*). The different shades of *red* within each archetype represent total deviation values. Each stimulus size III archetype was matched with a size modulation perimetry archetype with the highest cosine similarity.

**Figure 4.**
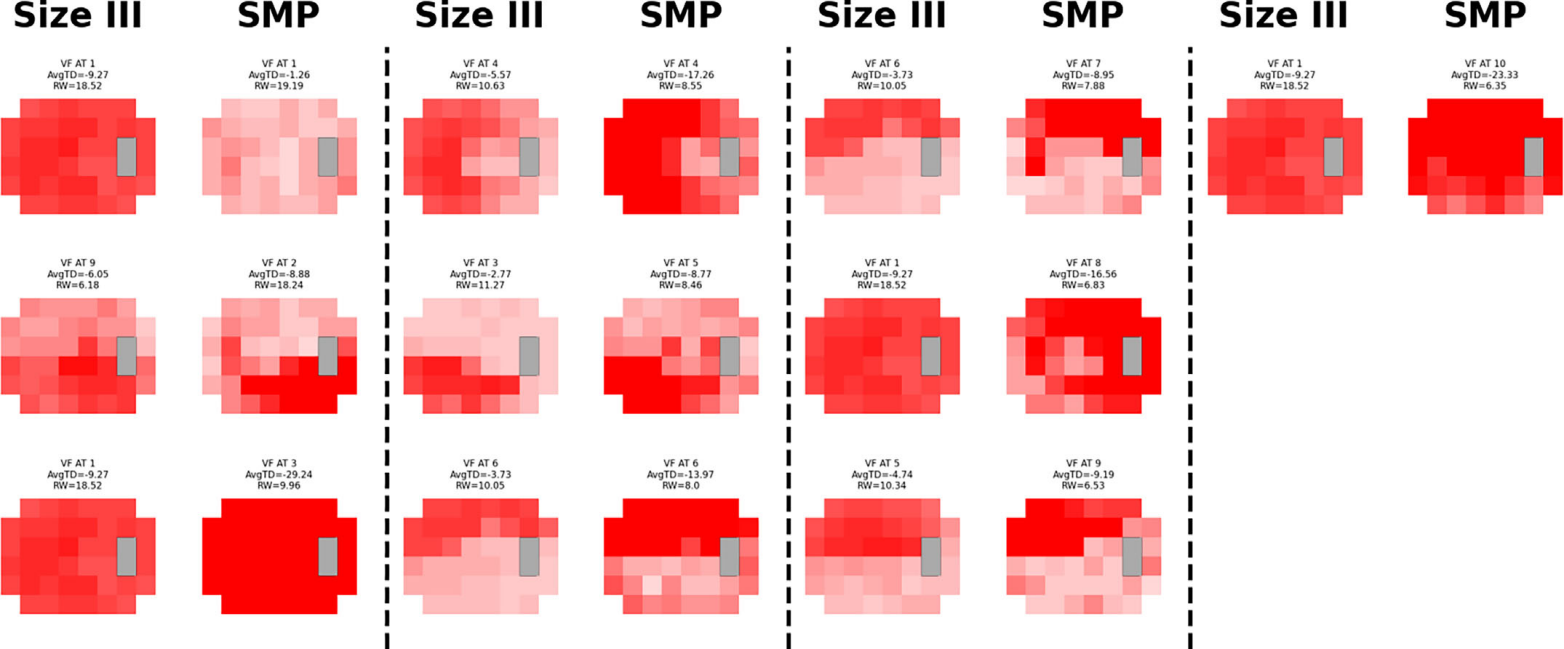
**AVFA SMP-size III mapping.** Visual field patterns for glaucoma using stimulus size III (*left*) and size modulation perimetry (*right*). The different shades of *red* within each archetype represent total deviation values. Each size modulation perimetry archetype was matched with a stimulus size III archetype with the highest cosine similarity.

The ATs for AVFA exhibited high cosine similarities (Figs. 3, 4; Table 1). In size III-SMP mapping, 9 of 10 size III ATs had a similarity score of ≥0.5, with 4 of 10 being highly similar (≥0.8). For SMP-size III mapping, all 10 SMP ATs had a corresponding size III AT with a similarity score of ≥0.5, and 4 of 10 ATs showed high similarity (≥0.8).

**Table 1.** **Absolute Visual Field Archetype Mapping.** Pairs of Archetypes by Visual Field Type Derived From Absolute Archetypal Visual Field Data for Glaucoma, With Corresponding Cosine Similarity Scores for Each Pair

Size III-SMP Mapping			SMP-Size III Mapping		
Size III	Mapped SMP	Cosine Similarity	Mapped Size III	SMP	Cosine Similarity
AT1	AT3	0.90	AT1	AT1	0.56
AT2	AT5	0.31	AT9	AT2	0.63
AT3	AT5	0.76	AT1	AT3	0.90
AT4	AT4	0.88	AT4	AT4	0.88
AT5	AT10	0.83	AT3	AT5	0.76
AT6	AT6	0.82	AT6	AT6	0.82
AT7	AT9	0.73	AT6	AT7	0.76
AT8	AT5	0.53	AT1	AT8	0.71
AT9	AT3	0.77	AT5	AT9	0.76
AT10	AT10	0.54	AT1	AT10	0.84

The leftmost side maps every single stimulus size III archetype to the size modulation perimetry archetype with the highest cosine similarity. The rightmost side maps every size modulation perimetry archetype to the stimulus size III archetype with the highest cosine similarity.

### Visual Field Change Analysis

**Figure 5.**
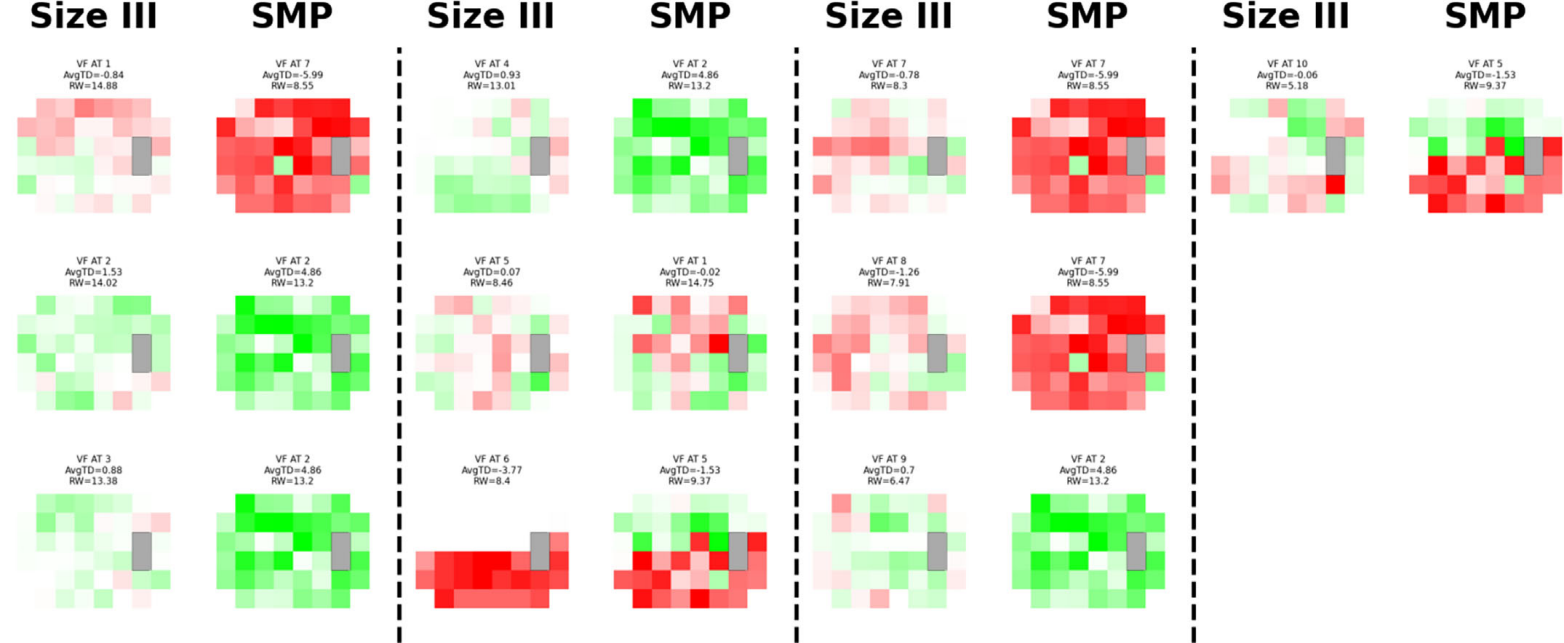
**VFCA size III-SMP mapping.** Visual field patterns for glaucoma using stimulus size III (*left*) and size modulation perimetry (*right*) over time with pointwise subtraction. The different shades of *red* and *green* within each archetype represent recovery and progression with total deviation value changes. Each stimulus size III was matched with a size modulation perimetry archetype with the highest cosine similarity.

**Figure 6.**
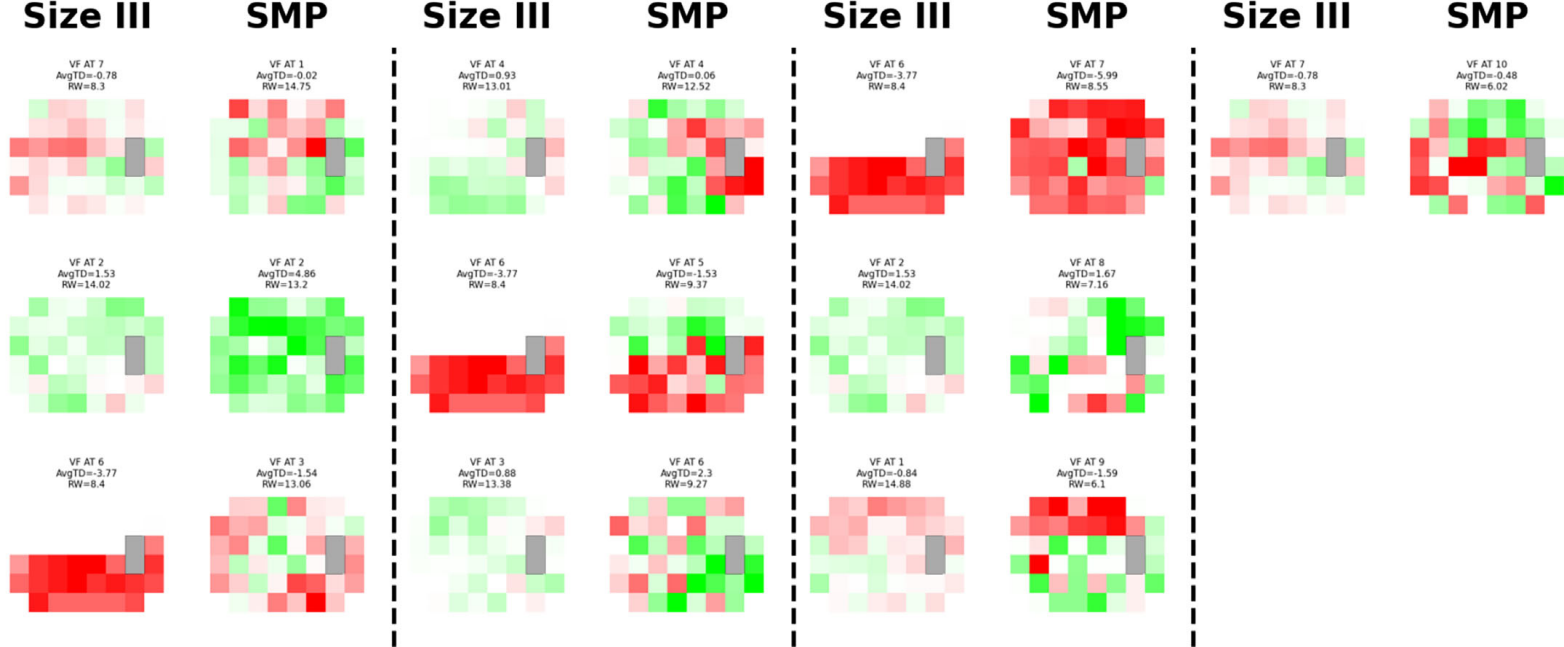
**VFCA SMP-size III mapping.** Visual field patterns for glaucoma using stimulus size III (*left*) and size modulation perimetry (*right*) over time with pointwise subtraction. The different shades of *red* and *green* within each archetype represent recovery and progression with total deviation value changes. Each size modulation perimetry archetype was matched with a stimulus size III archetype with the highest cosine similarity.

In size III-SMP mapping, only 3 of 10 ATs had a cosine similarity score ≥0.5, whereas SMP-size III mapping also had 3 of 10 ATs surpass this threshold. Despite these relatively low similarity scores, visual inspection of the ATs still revealed clear structural similarities. Size III AT1 and SMP AT9 both exhibit distinct superior arcuate defects, yet their cosine similarity score was 0.43 (Figs. 5, 6; Table 2).

**Table 2.** **Visual Field Change Archetype Mapping.** Pairs of Archetypes by Visual Field Type Derived From Archetypal Visual Field Data of Pointwise Change for Glaucoma, With Corresponding Cosine Similarity Scores for Each Pair

Size III-SMP Mapping			SMP-Size III Mapping		
Size III	Mapped SMP	Cosine Similarity	Mapped Size III	SMP	Cosine Similarity
AT1	AT7	0.45	AT7	AT1	0.32
AT2	AT2	0.61	AT2	AT2	0.61
AT3	AT2	0.54	AT6	AT3	0.35
AT4	AT2	0.34	AT4	AT4	0.26
AT5	AT1	0.31	AT6	AT5	0.67
AT6	AT5	0.67	AT3	AT6	0.25
AT7	AT7	0.37	AT6	AT7	0.52
AT8	AT7	0.45	AT2	AT8	0.31
AT9	AT2	0.34	AT1	AT9	0.43
AT10	AT5	0.26	AT7	AT10	0.34

The leftmost side maps every single stimulus size III archetype to the size modulation perimetry archetype with the highest cosine similarity. The rightmost side maps every size modulation perimetry archetype to the stimulus size III archetype with the highest cosine similarity.

## Discussions

We found that the patterns identified from AA for both size III and SMP VFs were highly consistent in AVFA and somewhat consistent in VFCA. These results suggest that despite differences in stimulus presentation, the underlying patterns of loss are similarly mapped between SAP and SMP. This also showcases AA's ability to consistently capture and quantify underlying disease characteristics.

The AVFA for SAP and SMP indicates that both modalities capture remarkably similar patterns in eyes with glaucoma. Whereas not achieving perfect similarity scores, values of approximately 0.5 still indicate meaningful pattern alignment. For example, size III AT8 and SMP AT5, which had a cosine similarity of 0.53, both clearly depict an inferior arcuate defect.

In contrast, the VFCA results were less definitive, likely due to two factors. First, the sample size was smaller as each is derived from a later VF subtracted from its baseline, effectively combining two VFs into one. AA performs best in large datasets where it can identify patterns within noise. Second, the intensity-blind cosine similarity struggles when VFs contain noise centered approximately 0 dB. If VF A alternated between 0.1 and 0 dB at each point while VF B alternated between 0 and 0.1 dB, their cosine similarity would be zero, despite both fields being nearly identical. This may explain why the AVFA yielded higher cosine similarities compared to VFCA, as most points in AVFA had a negative in TD.

Another disadvantage of cosine similarity is its sensitivity to differences in VF dynamic range, particularly in the presence of flooring. In our study, size III VFs were censored at a minimum sensitivity of 20 dB, whereas SMP fields were not. As a result, even when two ATs exhibited nearly identical visual defect patterns, differences in how low-sensitivity regions were represented relative to other parts of the VF within the dynamic range leads to lower cosine similarity scores. For example, AVFA size III AT3 and SMP AT5 appear to represent the same defect visually. However, because SMP has a deeper defect while the rest of the field remains similar, the cosine similarly was only 0.76 instead of approaching 1. Whereas censoring the SMP fields would improve cosine similarity scores, doing so would have negated the advantage of SMP in preserving a broader dynamic range.

Although SMP's signal-to-noise advantage may be most pronounced in early-moderate glaucoma, prior work^6^ shows that its variability remains flat in advanced loss. Our cohort therefore provides a stringent test of pattern concordance where SAP reliability is worse.

Ricco's area represents the diameter of the retinal patch within which ganglion-cell signals completely sum at a particular location. Detection can be improved equally by increasing the stimulus area by recruiting more ganglion cells, or luminance by increasing the response from those already stimulated, given the stimulus fits within a single cortical receptive field. Near the fovea, this diameter is small, and it widens with distance from the fovea and after ganglion-cell loss. SAP uses a fixed 0.43 degrees size III target, which often exceeds Ricco's area in healthy locations.^26^ In this scenario, the test operates under partial summation and modest increases in luminance can still lead to detection even after substantial RGC loss, reducing the effective signal. SMP, conversely, enlarges the stimulus on each trial until the spatial summation area (corresponding to a single cortical or multiple retinal ganglion cell receptive fields under SAP-like adaptation conditions) is engaged, ensuring all tests operate within complete summation.^27^ This avoids the flattening or underestimation of functional loss that occurs with SAP due to partial summation, leading to better detection of true retinal sensitivity loss. Although early disease is not represented in our dataset, this difference may be most relevant in such cases, and could also affect detection at the borders of moderate defects, with SMP potentially revealing larger or more complete defects than SAP.

Although our primary focus was on size III due to its widespread clinical use, we also generated absolute AT maps for SAP with sizes V and VI stimuli. These maps were qualitatively similar to those from size III. Whereas sizes III and V have been shown to be quantitatively very similar in moderate to severe glaucoma,^18^ this has not been formally demonstrated for size VI, although the observed patterns suggest a comparable spatial distribution of defects. In this disease range, sensitivity is often low enough that all three stimulus sizes are likely to operate within complete spatial summation. This may explain the similarity in patterns across stimulus sizes and suggests that spatial summation status alone does not account for the observed agreement.

Our study is not without limitations. We also used a normative database with 60 subjects tested twice to calculate TD values for SMP. Whereas this sample provided a useful reference, it may not fully encompass the variability present in a larger population. We selected 10 ATs to strike a balance between minimizing noise and preserving meaningful patterns, even if this was not the strictly optimal mathematical choice. We prioritized a lower AT count to reduce the number of “noise” ATs, although this came with the risk of potentially missing patterns. Across all models, the RSS curve remained relatively low, suggesting that increasing the AT count would not have provided significant additional benefit. Additionally, we observed some noise in the VFCA results, likely due to the smaller number of available fields. These fields are also not independent because each has the same baseline subtracted, which further reduces the number of unique observations. The cohort consisted mainly of eyes with moderate to advanced glaucoma (baseline SAP mean deviation approximately –6.7 dB), and it contained no eyes with early disease with no detectable field loss. As our dataset involved more severe cases of glaucoma, it is likely that both SAP and SMP operated within complete spatial summation, potentially contributing to the similarity in measured thresholds. We plan to separately test whether differences between the methods emerge in eyes with early damage, where partial summation may play a greater role.

This study underscores the stability of archetypal patterns despite variations in stimulus type and measurement constraints, reinforcing the potential of AA as a reliable tool for both clinical and research applications. Future studies could explore data harmonization techniques to better align pointwise VFs between SAP and SMP given their nonlinear relationship. Extending SMP similarity to other SAP stimulus sizes may further validate its generalizability. Clinically, this approach could be applied to quantify glaucomatous progression or recovery, enhancing diagnostic precision and enabling more personalized treatment strategies.

## Supplementary Material

Supplement 1
