# Supplementary material for: Archetypal Analysis Reveals Consistent Visual Field Patterns for Stimulus Size III and Size Modulation Perimetry in Glaucoma
Source: Transl Vis Sci Technol. 2025 Oct 10;14(10):12. doi: 10.1167/tvst.14.10.12 (PMC12524828; doi:10.1167/tvst.14.10.12)
Supplement: Supplement 1 [file tvst-14-10-12_s001.pdf]

Size III Absolute Archetype Map

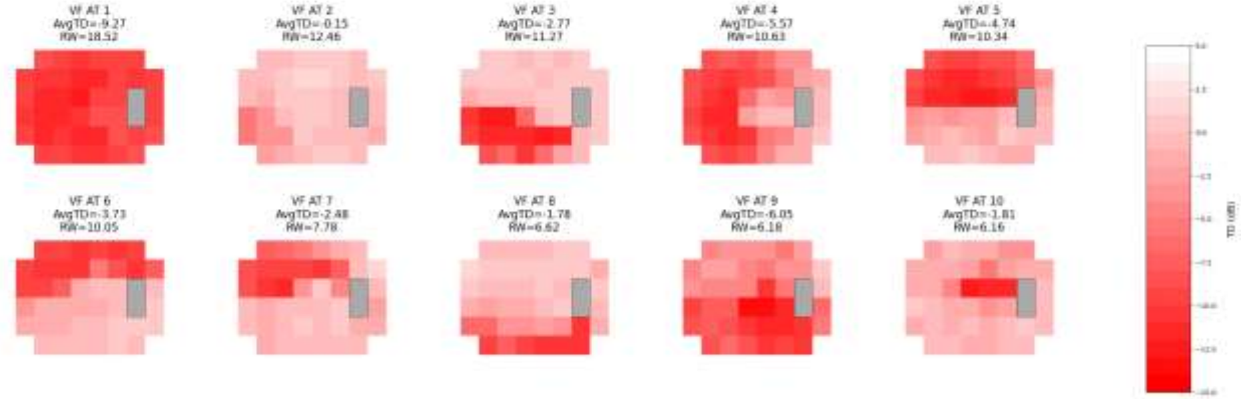

Supplemental Figure 1. Visual field patterns in glaucoma using stimulus size III, censored at 20 dB. Shades of red within each archetype represent total deviation values. The color scale ranges from -15 dB to 5 dB. Each archetype is displayed alongside its average total deviation value and relative weight as a percentage of the dataset. Archetypes are numbered and ordered by relative weight.

SMP Absolute Archetype Map

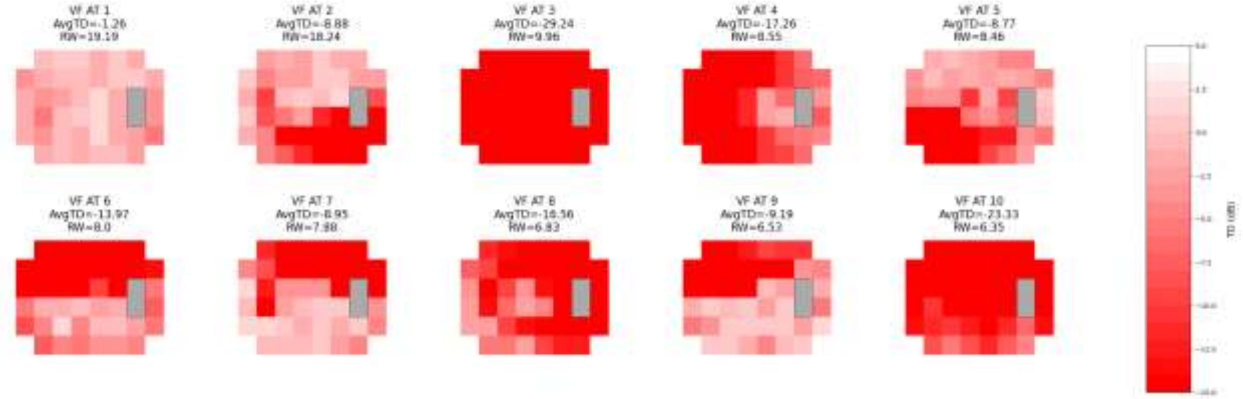

Supplemental Figure 2. Visual field patterns in glaucoma using size modulation perimetry. Shades of red within each archetype represent total deviation values. The color scale ranges from -15 dB to 5 dB. Each archetype is displayed alongside its average total deviation value and relative weight as a percentage of the dataset. Archetypes are numbered and ordered by relative weight.

Size III Change Archetype Map

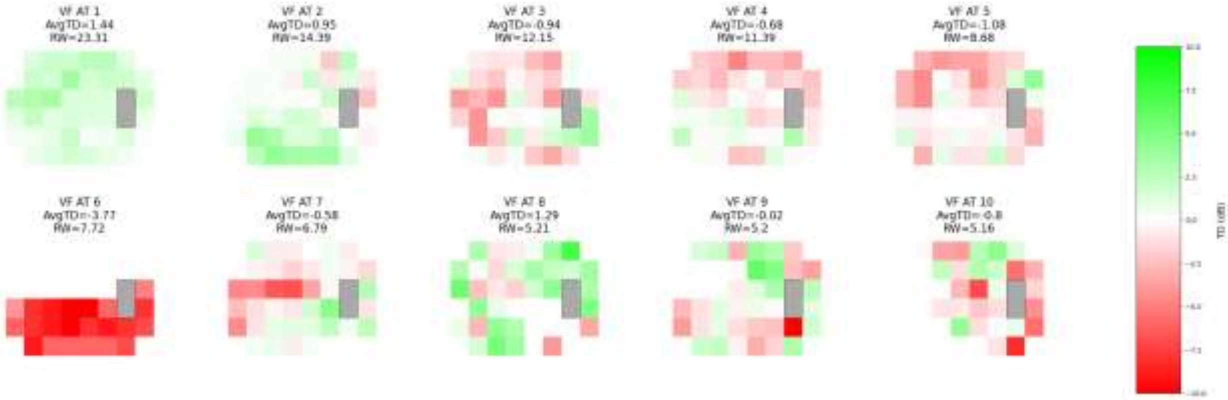

Supplemental Figure 3. Visual field pattern changes in glaucoma from baseline using censored stimulus size III. Shades of red and green within each archetype represent worsening or recovery in total deviation units. The color scale ranges from -10 dB to 10 dB. Each archetype is displayed alongside its average total deviation value and relative weight as a percentage of the dataset. Archetypes are numbered and ordered by relative weight.

SMP Change Archetype Map

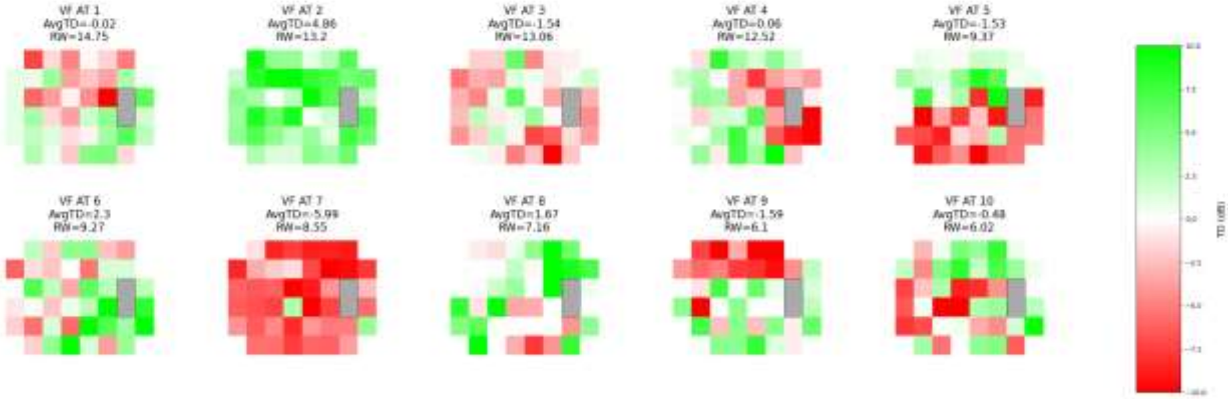

Supplemental Figure 4. Visual field pattern changes in glaucoma from baseline using size modulation perimetry. Shades of red and green within each archetype represent worsening or recovery in total deviation units. The color scale ranges from -10 dB to 10 dB. Each archetype is displayed alongside its average total deviation value and relative weight as a percentage of the dataset. Archetypes are numbered and ordered by relative weight.

Size V Absolute Archetype Map

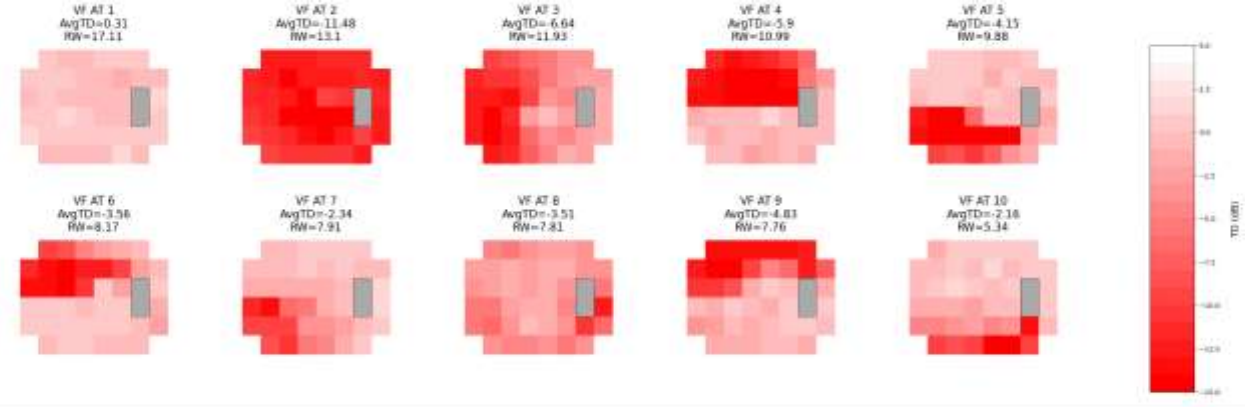

Supplemental Figure 5. Visual field patterns in glaucoma using stimulus size V, censored at 20 dB. Shades of red within each archetype represent total deviation values. The color scale ranges from -15 dB to 5 dB. Each archetype is displayed alongside its average total deviation value and relative weight as a percentage of the dataset. Archetypes are numbered and ordered by relative weight.

Size VI Absolute Archetype Map

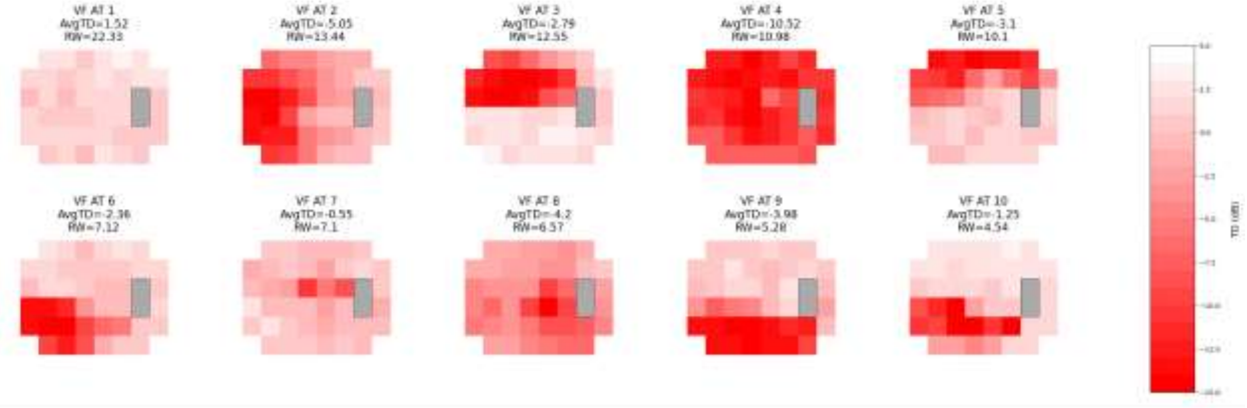

Supplemental Figure 6. Visual field patterns in glaucoma using stimulus size VI, censored at 20 dB. Shades of red within each archetype represent total deviation values. The color scale ranges from -15 dB to 5 dB. Each archetype is displayed alongside its average total deviation value and relative weight as a percentage of the dataset. Archetypes are numbered and ordered by relative weight.
